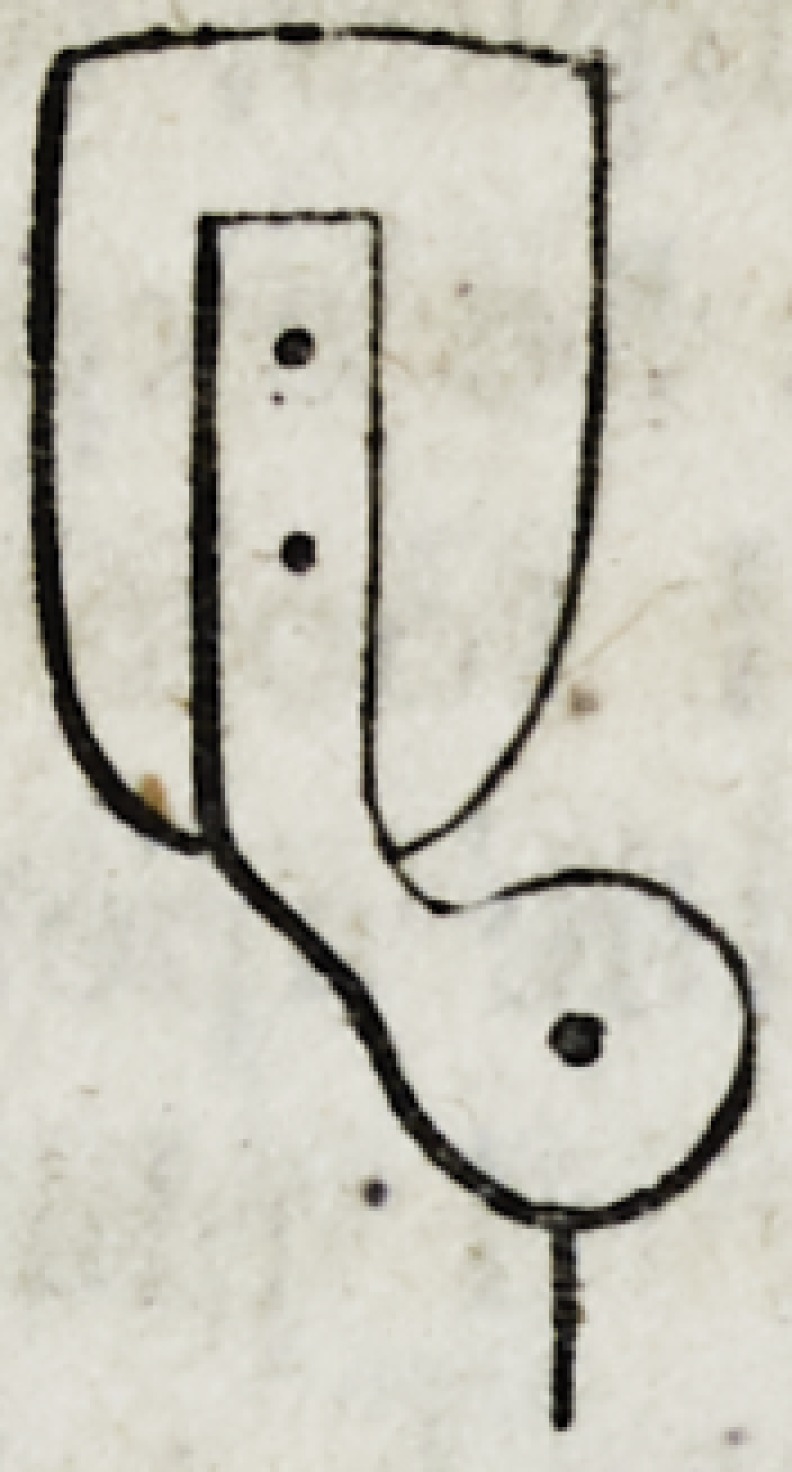# A Treatise on Mechanical Dentistry

**Published:** 1841-12

**Authors:** Solyman Brown


Vol. II.] DECEMBER, 1841. [No. 2.

### ARTICLE I.

*A Treatise on Mechanical Dentistry.* By Solyman Brown, M.D., D.D.S.

### CHAPTER I.

1. It is no part of the design of this series of articles, written at the repeated solicitation of many of my professional brethren, either to demonstrate the general utility of artificial teeth, or to persuade the community to procure them. The wants of the student in dentistry, will be consulted and met, as far as practicable, in the following treatise; and no pains will be spared by the author, in his efforts to collect and communicate in the plainest terms, all the practical instruction which the present condition of our art can supply.

2. Each section of this treatise will be numbered for the sake of convenient reference, and as many explanatory cuts will be given, as the nature of the subject may demand. If, in attempting to make the details of the art, perfectly intelligible to the student, the author should seem to the older members of the profession, to be too minute in his illustrations, they will no doubt on a moment's reflection, admit the validity of his excuse.

3. By Mechanical Dentistry is understood the art of constructing sets and parts of sets of artificial teeth; of inserting single teeth on roots or fangs; and of constructing artificial palates in cases where the natural organ is deficient.

A description of the method of proceeding in every supposable case will be the object of this series of papers.

4. The implements proper to the mechanical dentist will be minutely described as they become necessary in the progress of the work; it being premised that a bench, hammers of various kinds, an anvil, vice, files, &c. must be understood to be essential.

5. It being the opinion of the author, founded on a settled conviction resulting from observation and experience, that the necessity of using human teeth, blocks of ivory, or other animal substance, is wholly superseded by the great improvements which have been recently made in the manufacture of incorruptible, mineral teeth; the directions given in this essay will relate exclusively to the latter, which he hopes every honourable practitioner will endeavour to introduce into his practice, to the exclusion of all substances which are liable to be decomposed by the fluids of the mouth, lose their beauty, and infect the breath with loathsome poison.

*Of the Insertion of a Single Tooth on the natural root.*

6. As broken parts of the crown often remain attached to the root, a pair of excising forceps becomes necessary. This instrument should be about five inches in length, having the cutting edges one-tenth of an inch in breadth.

In this wood cut (*a*) represents the forceps, and (*b*) the breadth and shape of the cutting edges. These forceps should be of sufficient strength and material not to bend or spring under the pressure of the hands, and should be used with such gentleness as not to strain, shake, or otherwise injure the root.

After the prominent parts of the crown shall have been removed by means of the forceps above described, as nearly as possible to the semi-circular arch of the gum, a round, elliptical or half round file is employed to complete the process.

a b c

The file should be fine-cut, and ought to be used with delicacy, as well to save the sensibilities of the patient, as to avoid agitating the root to such a degree as to produce inflammation of the investing membrane and surrounding structure.

7. When the file has reduced the root to the edge of the gum, and even a little beyond it, the next operation is to perforate the fang, by enlarging the natural nervous tube by means of a broach or hand drill of something like the following construction.

The point may be either square, or pentagonal like the common watch-maker's broach, and should be of sufficient size to produce an orifice capable of receiving a pivot of the size of a large knitting needle. Several broaches of smaller sizes are necessary to enlarge the natural orifice by degrees to the required diameter. The depth of the orifice should be one-fourth of an inch, in roots that will admit of it.

8. Select a mineral tooth as nearly as possible of the colour of the adjacent teeth, taking care that the shade never be lighter than that of the natural teeth in the same mouth. Let it be moreover of the proper length, breadth, and thickness, with as little grinding as possible. Some change of shape and dimensions, is however generally necessary, for which a suitable grinding apparatus is indispensable. Machines for grinding mineral teeth are of various kinds, of which a common grindstone is the most obvious and simple. Small and portable stones for the use of dentists, are prepared and mounted by dental instrument-makers, of which the following is a draft:

An improvement on this construction has been attempted by Messrs. Royce & Esterly, ingenious dentists, resident in Poughkeepsie, in the state of New York. This grinding apparatus consists of simple system of cog-wheels by means of which one of the two stones has an accelerated motion. This patent has the advantage of elegance of structure, neatness in the operation, and the easy change of stones of various dimensions; and is moreover well adapted to the use of a newly invented grinding wheel, which will be described hereafter.

The two foregoing machines for grinding teeth, are well adapted to the use of travelling dentists; but for those members of the profession who are stationary, foot lathes are much the most efficient. These may be of very simple construction, thus:

Lathes constructed on this principle may be enclosed in ornamental cases, and thus become articles of furniture not unbecoming the operating room of the genteel dental surgeon; but they are generally better fitted to the laboratory of the artificial workmen.

Of the stones or wheels employed in grinding mineral teeth, many varieties are in vogue. Some good workmen use wooden wheels about six inches in diameter, the circumferences of which are turned into convenient forms, and covered with hot glue and emery. These are commonly called emery wheels, and have been long used in the mechanic arts.

Many dentists still adhere to the use of grindstones of small dimensions, but from recent experiments I am led to prefer a composition wheel made of shellac and emery, combined in certain proportions when hot, afterwards cooled in moulds of strong metal provided expressly for the purpose, in which the material is compressed by great mechanical force.

10. The evident advantages of this species of grinding apparatus are, first, its little liability to change of form from constant use: secondly, its capability of retaining sharp edges, corners and curves, longer than any other substance hitherto employed ; thirdly its economy, being exceedingly durable, and fourthly, the mechanical correctness of its execution, arising from its hardness and solidity.

The following are some of the convenient forms of the cutting edges of these wheels, as they come from the moulds.

Although these edges, corners, planes and curves remain long with little apparent change, which is by no means true of any other wheels used for the purpose excepting copper ones; yet it must be evident that constant use will more or less change those original forms unless the mode of grinding upon them shall be calculated to preserve the primitive shapes; a point to which ordinary ingenuity will direct the attention of the artist.

11. As the maufacture of these wheels is attended with considerable expense, in the way of moulds and presses to give form and compactness to the materials used, it is presumed that dentists will prefer to purchase rather than to make them, and therefore any further description is deemed superfluous.

When the tooth has been carefully and accurately fitted to the stump or root by grinding, and made of the proper length, breadth and thickness, it may be fixed temporarily in its place with a pivot of white pine, poplar or other soft wood, for the purpose of affording the patient an opportunity of suggesting any alteration that may be desired.

12. When all parties are fully satisfied with the appearance of the artificial substitute, and when the antagonizing teeth in the opposite jaw meet with no obstruction on closing the mouth, the tooth is ready to be permanently fixed in its place; but before proceeding to establish it firmly on its pivot, an instrument may in some cases be employed with advantage to cut away the central portions of the root in order to make a close fit of the crown to the stump.

*a*

When the point (*a*) of this instrument is inserted in the orifice of the root, a rotary motion files away the parts proper to be removed in order to perfect the joint.

By placing eight or ten thicknesses of gold foil between the crown and root, the fluids of the mouth may be wholly excluded.

13. The pivot which is to sustain the new crown in its position, should be made of the best of hickory, as no other wood proper to the American soil, possesses so much strength and elasticity combined. Force the pivot, when properly rounded and smoothed with a file, into the orifice of the artificial crown, and cut the part of the pivot which projects from the crown to such length as the orifice in the root will admit; after which the size of the projecting portion of the pivot must be adjusted to the orifice of the fang. The force used to insert the pivot in the root need not be great, inasmuch as the swelling of the wood when saturated with moisture and heated to the temperature of the body, will secure it firmly in its place. The following are a front and a lateral view of a crown when thus prepared for insertion.

14. In some instances the cavity in the natural root has become by decay, too large for a pivot of the description just given, in which case pivots of gold or platina of a square or flat form made jagged at the corners, must be substituted for the hickory, as follows: First fill the enlarged orifice of the root with a plug of soft wood, which must be trimmed neatly to correspond with the end of the fang. Then insert a similar plug into the mineral crown, into which plug screw the end of the metallic pivot after it has been passed through one or more of the holes of the following screw plate.

It will be evident that the metallic pivot just described, will be round at one end and square or flat at the other, thus:

*a*  *b*

The end (*a*) is inserted into the natural root, and the end (*b*) into the artificial crown.

15. The final adjustment of teeth set in either of the methods just described, may be effected by the aid of a wooden instrument somewhat of the form which follows, and should be made of very tough and soft wood.

If the strength of the hand should be found insufficent to force the pivot perfectly into its place, a slight blow on the end of the wood, when placed upon the end of the tooth, will complete the operation.

16. It sometimes happens that the position of the root is such that a crown placed upon it as above described, will not occupy the desired position in the mouth, being too far back or forward, or having too great a lateral inclination to the right or left. In these circumstances an entirely new course must be pursued; another kind of mineral crown must be procured known as a *plate tooth,* in contradistinction to the former kind called a *pivot tooth.* The plate teeth are capable of being attached firmly to a metallic plate, and as gold is by far the best metal for this purpose, I shall denominate it throughout this treatise, a *gold plate.*

In a case requiring to be treated in this manner in consequence of some unnatural condition of the root or of the adjoining teeth, after filing and preparing the root exactly as before, take common beeswax and bring it to a pliable consistency by steeping it for a few minutes in warm water. When in the state of stiff dough or putty, place the wax in a frame made of tin, German silver, or other convenient metal, conformed to the arch of the jaw, as follows:

Of these frames the operator should be provided with various forms and sizes to suit mouths of different dimensions.

17. When a frame of the proper size and curvature has been filled with the softened wax, insert it cautiously into the mouth of the patient, and press the wax gently but effectually against the cutting edges of the teeth adjacent to the space to be filled, until the teeth and gums are completely imbedded in the wax, which should be pressed against the gums both inside and out, by the thumb and fingers of the operator, holding the frame firmly in its proper position with the other hand. The frame and wax are next to be cautiously withdrawn from the mouth in such manner as not to mar in the least the impression thus obtained. Let the wax cool, and then oil it with a soft brush and olive oil slightly; after which thrust a common pin or a small wire into the centre of each cavity made by the teeth in the wax, in order to render the plaster-cast taken from the wax less liable to injury.

Surround the wax thus prepared with a ribbon of paper two inches in width, secured to the wax by pins or twine, and then set the whole in a vessel of sand which will secure the paper in its place.

18. Take calcined plaster of Paris or gypsum, ground and sifted, and mix with it a sufficient quantity of water to reduce it to creamy paste, the exact consistency of which is best determined by taking in a small vessel as much water as would nearly fill the wax mould and the paper rim, and dropping into it little by little as much superfine plaster as shall just absorb all the water, then stir the mixture for a moment and turn it into the mould.

After fifteen or twenty minutes, as must be determined by the quality of the plaster, and the lapse of time since its calcination, the paper may be removed, and the wax together with the plaster-cast immersed in warm water until they can be separated with ease and safety. Trim the cast with a sharp knife, being careful to give the whole such a form as will be easily withdrawn from the metal which is to be cast upon it. Dry the plaster thoroughly and it is ready for use.

It is always best and sometimes necessary to take two similar casts in the manner just described, for distinct purposes, as will be seen hereafter.

19. Some good artificial workmen take their metallic castings in sand, after the manner of brass and iron founders, but I have found the following process to be much more direct and explicit, and every way successful. Take an iron ladle of hemispheric shape, and capable of holding at least a pint and a half of fluid; melt in it as much lead as will nearly fill the ladle. Into this molten lead immerse one of the plaster-casts, and depress it by laying weights upon it till the points of the teeth shall be sunk about one inch below the surface of the melted lead in the ladle. When cool, immerse the whole in water, and carefully remove and wash away the plaster. Cover the surface of the lead with which the other metal is to come in contact, either with the smoke of a lamp, or with *whiting* laid on wet with a brush, in order to prevent the adhesion of the melted tin which is to be poured into it.

Dry the leaden mould well, and place it on a vessel filled with water or wet sand, so that the lead shall be sunk in the water or sand about an inch, to prevent its fusion when the tin shall be poured into it. Surround the impression of the teeth in the mould with a rim of tin, copper, or brass, an inch or more in width, as in the following cut:

(*a*) Denotes the metal ring. (*b*) Denotes the leaden mould. (*c*) Denotes the vessel with water or sand.

Fill the rim (*a*) with melted tin just at the point of fusion, or at such a temperature that it will not char dry pine chips or shavings; at any temperature higher than this there will be danger of fusing the lead, and thus of uniting the metals into one mass. But inasmuch as the tin fuses at a lower heat than lead, and as the cold water or wet sand will maintain the low temperature of the lead, there will be no danger of spoiling the cast, in case the foregoing rules are carefully regarded. The two metals when cool must be separated by means of a heavy hammer, assisted when necessary with wedges of iron. The two parts of the mould are now ready for use. Experience will soon instruct the beginner so to trim the plaster-cast, as that the tin may be withdrawn easily from the lead.

20. The operator is now provided with metallic casts similar to those which he will be frequently called upon to take in the exercise of his art, and between which his plate is to be struck.

Let him then in the next place, cut a piece of gold plate of the thickness of a smooth shilling into such shape as exactly to occupy the space on the tin cast in which the tooth is to be inserted. When the gold plate is adjusted in its position, and bent by the aid of a hammer or pair of pliers nearly into the shape desired, bring the tin and lead casts together upon it, and with a smart blow with a heavy sledge-hammer, force the two metals into close contact, by which means the gold plate is swedged into the exact shape of the parts to which it is to be applied.

This latter operation is sometimes performed by means of a powerful vice which perhaps effects the object with equal certainty.

21. Separate the casts once more, and place the gold plate on the second plaster-cast, which has been reserved for this part of the process. If the casts were equally perfect, the plate will fit the plaster casts as it did the tin; and perhaps I may as well mention it here as any where, that if the operator wishes his plaster-casts to be clean and hard, it should be made warm and smeared with two or three coats of boiled linseed oil, applied in quick succession by means of a common paint brush, and then suffered to dry.

22. A plate tooth must now be selected of proper color, form and size, and having platina pivots inserted during the process of its manufacture to attach it to the plate. At this point the operator finds the necessity for two implements of the description following:

*a*

*b*

The shears (*a*) must be strong; and the plate-punch (*b*) of sufficient material to sustain the force required to perforate the plate without springing or breaking. With the use of these instruments together with a half round file, cut a plate of gold nearly as large as the back surface of the tooth; punch the holes as required, and insert the pivots as in this cut.

23. The process of soldering the tooth to the plate, must now be understood. To this end, procure a soldering lamp of some thing like the following form.

The orifice of the spout into which the wick is inserted, should be at least three-fourths of an inch in diameter, and the vessel capable of containing a quart or more of oil.

24. A large blowpipe sixteen inches in length must next be procured, having a large orifice, and yet capable of bringing a jet of flame to an exact focus when required. The long blowpipe presents a too great exposure of the eyes of the operator, and a pair of green glass goggles contribute to the same desirable result. Before lighting the lamp, cover the surface of the tooth on every side excepting that on which the plate is fixed, with a coating of plaster or Spanish white and water, and when time will permit suffer the coating to dry before exposing it to the blowpipe.

25. The surface of the gold plate already slightly rivetted to the tooth, must be washed with a solution of borax (subborate of soda) ground with clean water, and laid on with a camel's hair brush, and care must be taken that every part of the work where it is desired that the solder should take effect, should be touched with the solution. The best surface on which to grind or rub the borax is a common slate, such as children use at school.

The solder employed with plate twenty carats fine, may be prepared as follows: To one English sovereign or half an American eagle, add eighty grains of silver and forty grains of copper. Melt once with borax. In order to fit it for convenient use, it should be rolled into thin plate and well cleaned.

26. As some students may desire to understand the process of manufacturing their own plate in cases of necessity; Take an English guinea or sovereign, or an American half eagle, and alloy it with an American five cent piece of silver, which will reduce it to something less than twenty carat gold. Melt the two metals in a crucible with a small lump of borax and when cast into an ingot, hammer and roll for use. After being rolled the plate should be annealed by heating it to redness; and then cleaned by boiling in sulphuric acid diluted with water.

27. The foregoing hints are deemed sufficient to guide the student in the preparation of his own solder and plate when compelled to prepare them for himself; but as a rolling mill and other implements are necessary to this course, dentists generally procure their plate and solder ready prepared.

28. In submitting a piece of work to the action of the blowpipe, a piece of charcoal, cork or pumice stone is used to sustain the work and confine the heat. Charcoal is most readily obtained in masses of convenient size, but a large and flat piece of cork, or a block of pumice stone answers nearly the same purpose, and on some accounts are frequently preferred. When soldering whole sets of teeth or large pieces, a round block of charcoal four inches in diameter and six inches in length, hollowed in the form of a mortar, and having the outer surface covered with plaster laid on smoothly with a knife or trowel, will be found very useful and durable.

29. When first bringing the flame of the soldering lamp upon a mineral tooth under the blowpipe, care must be taken to raise the heat slowly, lest a too sudden expansion of the surface of the enamel should crack it, and render the tooth useless; and a similar caution should be used when cooling the teeth after the operation of soldering has been completed.

30. The skilful use of the blowpipe requires that a constant blast should be given till the solder fuses; or at any rate the interruptions should be few as possible. To this end the operator must acquire the habit of breathing through his nostrils while a stream of air is constantly projected from the blowpipe by the action of the muscles of the cheeks, which may be made to expel a current of air from the mouth even during the act of inhalation through the nostrils. This process requires some practice, but may be effectually acquired by determined perseverance, without it the process of soldering can be but imperfectly effected.

Doctor Black's self-acting blowpipe in which the burning steam of alcohol is used instead of oil, may be managed successfully by a careful workman after a little practice, but the heat being very powerful exposes the gold to fusion. For general use the soldering lamp just described will be found the most safely available to the mechanical dentist.

31. When the piece to be soldered has been brought to redness, the operator should watch the progress of his work carefully so as to be ready to remove the piece from the flame the moment the solder fuses and spreads as desired over the surface of the gold. This should be done for two reasons, first, that the plate itself may not be melted as well as the solder; and secondly, that the solder may not be too much dissipated by an excess of heat. The great art of soldering consists in knowing just the quantity of flame proper to be used in a given case, and the exact point of time when the heat should be withdrawn.

After the piece has become entirely cool, it may be placed in an earthen vessel containing equal parts of water and sulphuric acid. Indeed any *simple* acid may be used with safety and success, taking care that nitric and muriatic acid be not used together, for this mixture constitutes the *aqua regia*, which is the only sure solvent of gold.

The action of the diluted acid will be accelerated by heat, which requires a copper vessel: but if the water has been recently mixed with the acid, sufficient heat will be evolved by the chemical union of the two fluids, to accomplish the purpose of cleaning the gold in a few moments.

32. The surface of the solder may now be rendered smooth by the use of files, when the tooth and its plate will be ready to be united; but before this is done, a gold or platina pivot must be inserted in that precise point of the plate which corresponds with the orifice in the root of the tooth, and this pivot should be square or flat as described in section 14. When this pivot has been soldered into the plate so that it will exactly fit its place in the mouth, adjust the plate to the second plaster-cast so that it will fit it as it does the place in the mouth where it is to be permanently worn. Then with files and the grinding apparatus fit the tooth to the plate, and give it the exact position desired as regards the adjacent teeth, and those in the opposite jaw. Support the tooth when rightly adjusted, by means of plaster of Paris mixed with equal parts of common sand, and applied in a state of paste to the surface of the tooth and the adjacent parts of the plaster-casts. The sand has the effect of preserving the plaster from cracking when heated by the blowpipe.

33. The piece is now ready when well dried for its final soldering; and as a great quantity of heat will be required to bring the plaster-cast to the temperature necessary for fusing the solder, the whole may be heated to redness in a ladle over a fire; or the concave piece of coal before described may be used for confining the blast of the blowpipe.

34. Use a liberal quantity of solder in joining the backs of the teeth to the principal plate, in order to give both strength and beauty to the work.

Indeed this is a part of the work which should never give way, because there is always room in this sharp angle to use a quantity of material which shall secure it from fracture.

35. If the work has been properly conducted thus far, all that part of the plate not covering the end of the natural root, and connecting with the tooth, may be filed away; and then the whole should be polished on a lathe similar to that which has been described as a grinding apparatus in section 8, by means of a brush wheel, as follows:

Common sand, ground pumice stone, emery, the *debris* of a grindstone, or crocus, may be used on the brush with water, and the final polish may be given with a similar circular brush and dry whiting.

36. In case the tooth requires to be set farther forward than could be done on a wooden pivot, the piece constructed as already described, will have the following appearances when viewed laterally and from behind.

When the pivot requires to be placed on one side of the centre of the tooth, it may assume the following appearance.

As metallic pivots are liable to work loose when inserted in the naked bone of the root, a covering of floss silk or even of raw cotton wound upon the pivot, will have the effect of preventing the friction of the bony structure. Or in some instances when the size of the root will allow it, a plug of soft wood may be first inserted, into which the metal pivot may be gently driven.

In concluding my directions for inserting a single mineral tooth upon a natural root, it becomes necessary to apprise the student of the propriety of so placing and shaping the artificial crown, that it may not come in contact with the teeth of the opposing jaw, inasmuch as constant agitation would soon either break or loosen the pivot, and thus render the work useless.

[to be continued.]

## Figures and Tables

**Figure f1:**
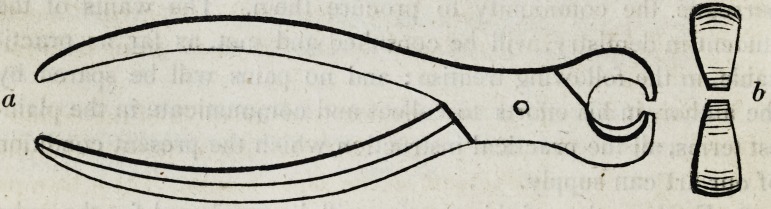


**Figure f2:**
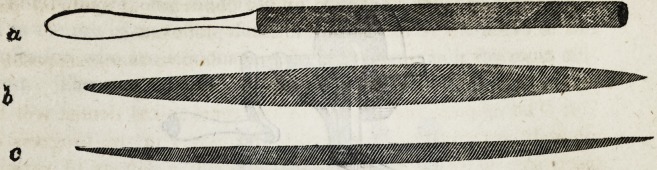


**Figure f3:**
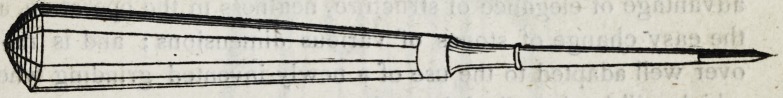


**Figure f4:**
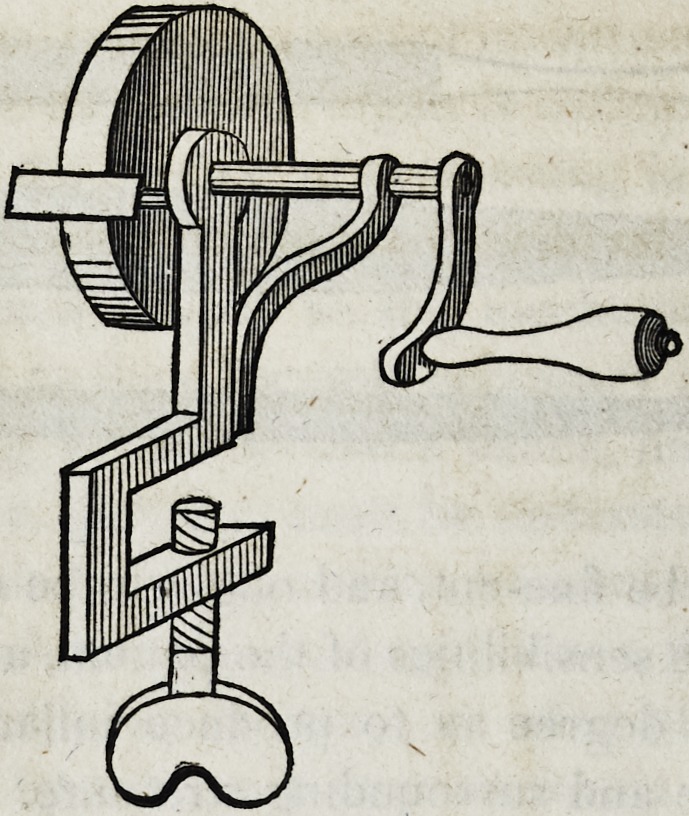


**Figure f5:**
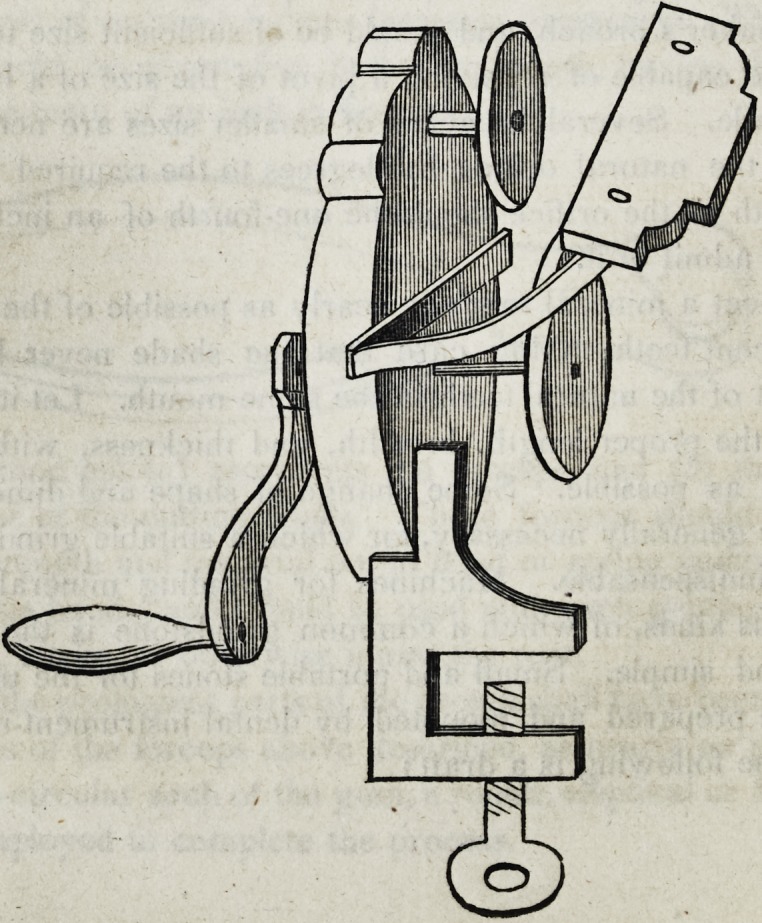


**Figure f6:**
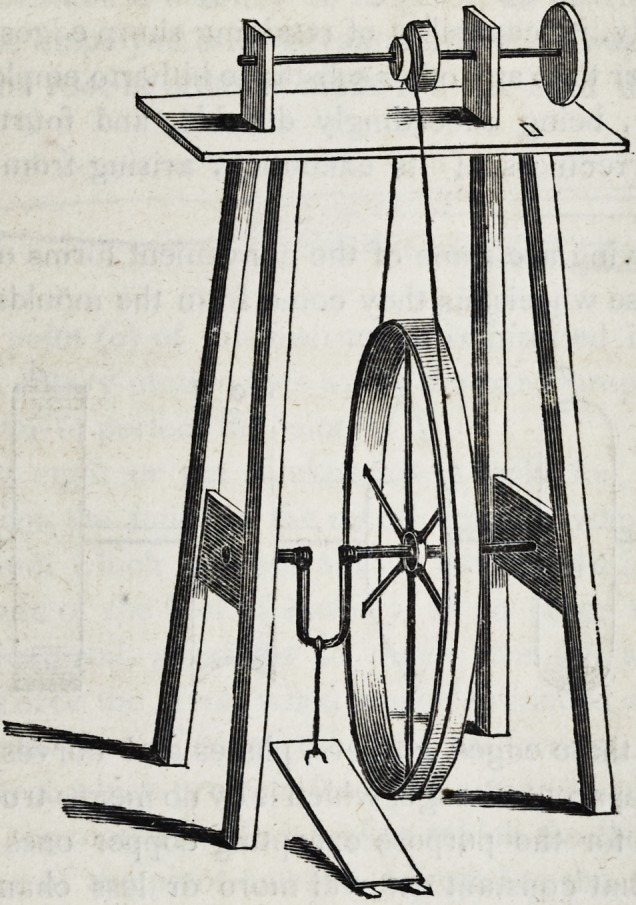


**Figure f7:**
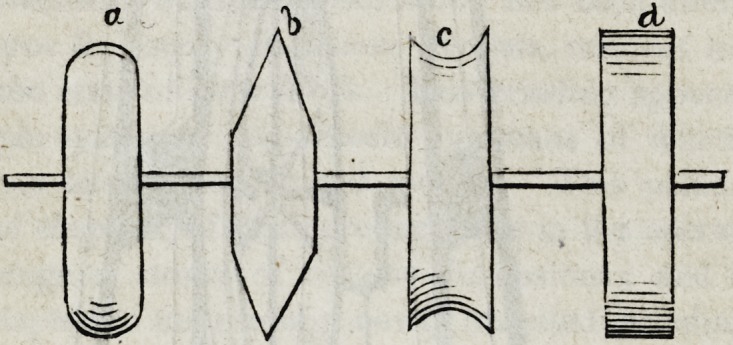


**Figure f8:**
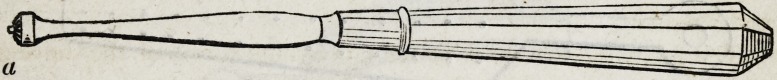


**Figure f9:**
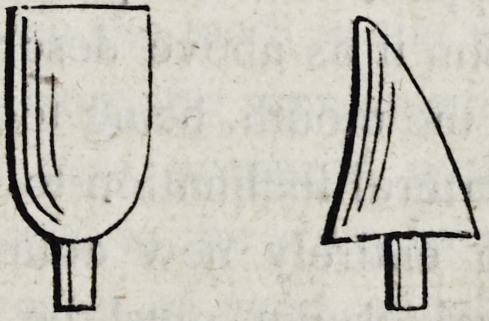


**Figure f10:**
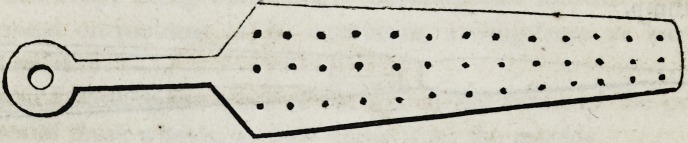


**Figure f11:**
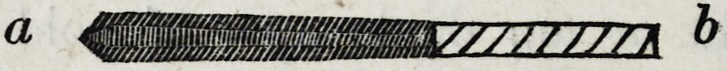


**Figure f12:**
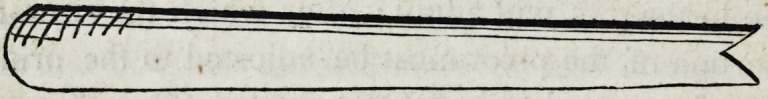


**Figure f13:**
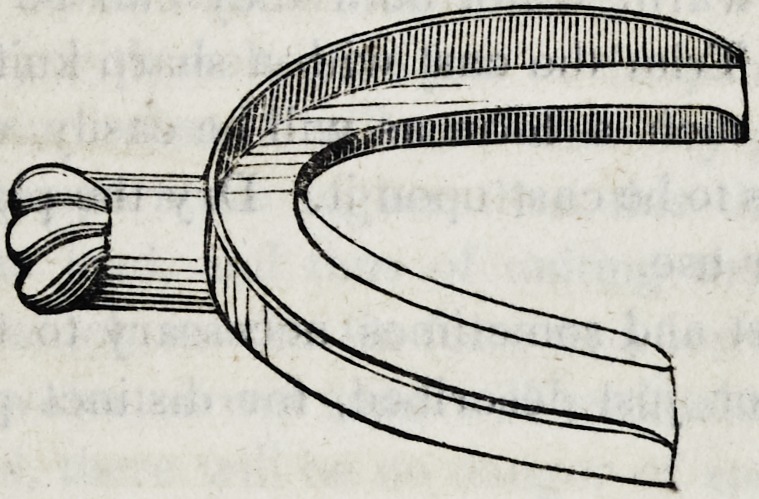


**Figure f14:**
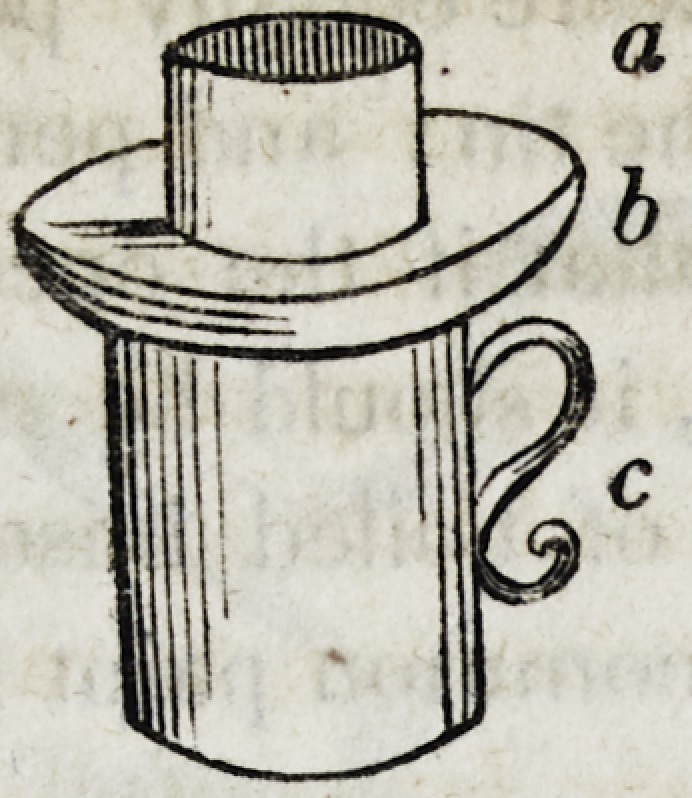


**Figure f15:**
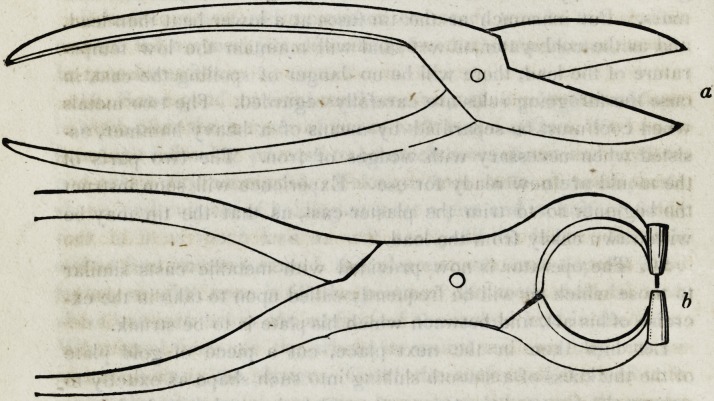


**Figure f16:**
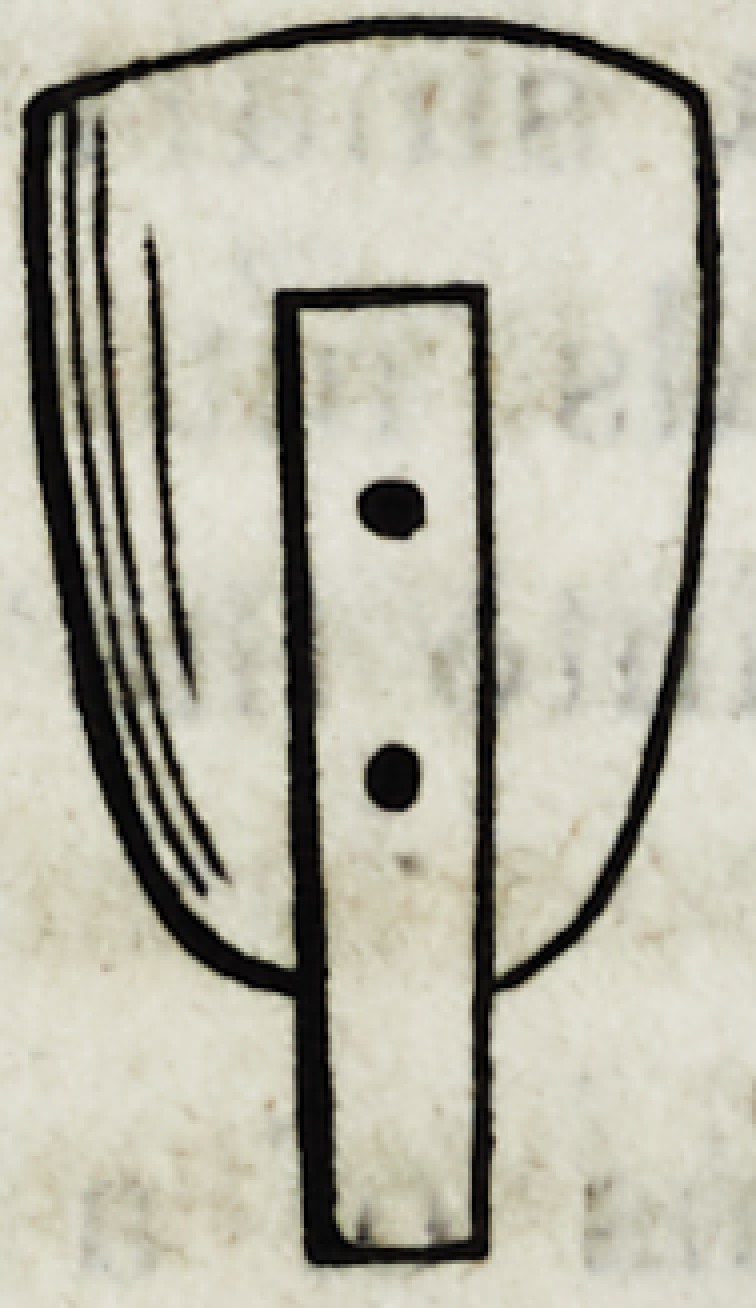


**Figure f17:**
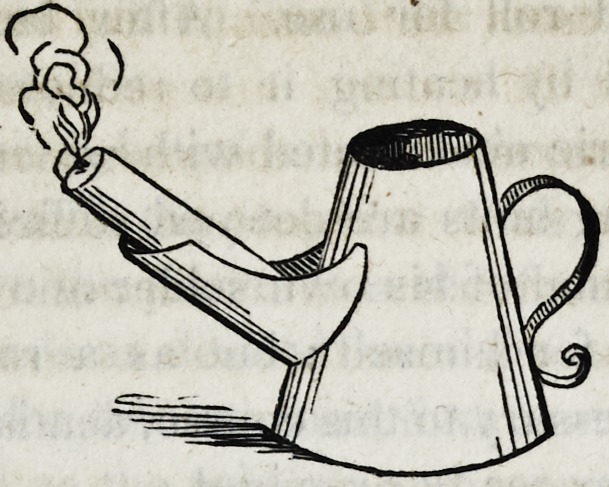


**Figure f18:**
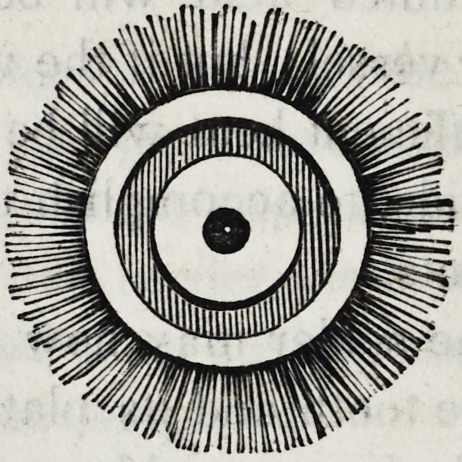


**Figure f19:**
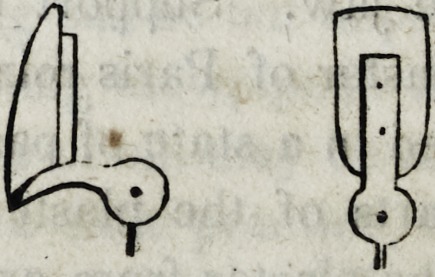


**Figure f20:**